# Interstitial Pneumonitis Related to Pegylated Interferon Alfa-2a Treatment in a Patient with Chronic Hepatitis C

**DOI:** 10.5005/jp-journals-10018-1176

**Published:** 2016-07-09

**Authors:** Erhan Alkan, Mete Akin, Haydar Adanir, Yasar Tuna

**Affiliations:** 1 Department of Gastroenterology, Burdur State Hospital, Burdur, Turkey; 2 Department of Gastroenterology, Akdeniz University School of Medicine, Antalya, Turkey; 3 Department of Gastroenterology, Diyarbakir Training and Research Hospital Gastroenterology Clinic, Diyarbakir, Turkey; 4 Department of Gastroenterology, Akdeniz University Faculty of Medicine, Antalya, Turkey

**Keywords:** Chronic hepatitis C, Interstitial pneumonitis, Pegylated interferon, Therapy.

## Abstract

Alkan E, Akin M, Adanir H, Tuna Y. Interstitial Pneumonitis Related to Pegylated Interferon Alfa-2a Treatment in a Patient with Chronic Hepatitis C. Euroasian J Hepato-Gastroenterol 2016;6(1):91-92.

Dear Editor,

Despite the newly developed oral antiviral treatments, pegylated interferon (PEG IFN) alpha still has an important place in the treatment of chronic hepatitis C in many countries and in the treatment of chronic hepatitis B in selected cases. Pegylated interferons have certain side effects, primarily flu-like symptoms, myalgia, fatigue, gastrointestinal disorders, psychiatric disorders, and hematological disorders.^1^ In this article, the interstitial pneumonitis that developed during PEG IFN alpha-2a treatment in a patient with chronic hepatitis C is reported as a more rare and serious side effect.

The treatment of PEG IFN alpha-2a 180 µg/once a week and ribavirin 1000 mg/day was initialized for a 46-year-old female patient with the diagnosis of chronic hepatitis C, genotype 1b. Hepatitis C virus (HCV) Ribonucleic acide was < 15 copy/mL in the 12th week of the treatment and negative in the 24th week. The patient applied to the gastroenterology clinic with the complaint of dry cough and shortness of breath in the 28th week of the treatment. The patient did not have a history of pulmonary disease. Physical examination revealed bilateral crepitations at the lung bases. The laboratory values in this period were normal apart from the hemoglobin of 11.2 gm/dL. The department of chest diseases was also consulted. Bilateral infiltrative appearance being prominent in the lower lobe of the right lung was detected in the chest radiography (Fig. 1) and high-resolution torax tomography demonstrated crystallization in the interlobular septates in the lower lobe of both lungs and increase in ground glass attenuation (Fig. 2). There was a decrease in the restrictive pattern and diffusing capacity in the pulmonary function tests. Infectious etiology was not detected in culture and bronchoalveolar lavage fluid analyses. It was thought that the current situation of the patient is interstitial pneumonia due to the use of IFN. The treatment was ceased and the patient started to be monitored. The clinical condition of the patient gradually improved after the discontinuation of the treatment. Hepatitis C recurrence was observed in the follow-up; however, significant lung symptom was not observed.

**Fig. 1:**
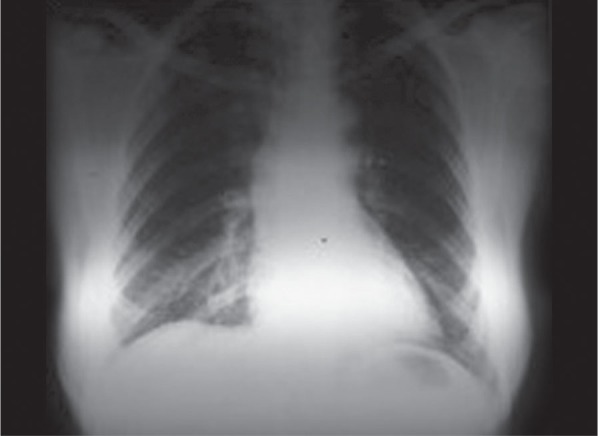
The chest radiography shows bilateral infiltrative appearance being prominent in the lower lobe of the right lung

**Fig. 2:**
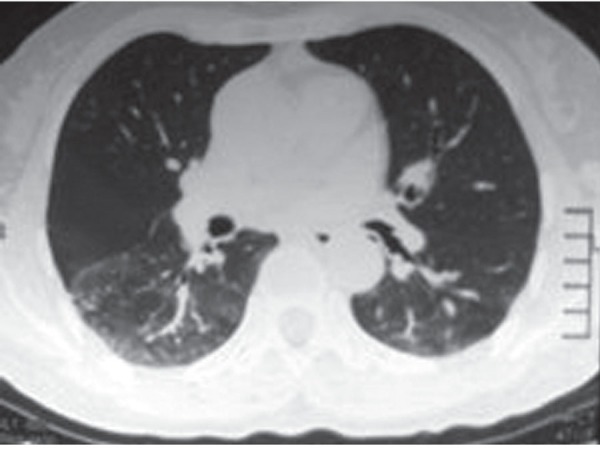
High-resolution torax tomography demonstrates crystallization in the interlobular septates in the lower lobe of both lungs and increase in ground glass attenuation

Pulmonary toxicity is a rare condition observed in patients with HCV treated with the combination of PEG IFN and ribavirin. Interstitial pneumonia, bronchiolitis obliterans with organizing pneumonia (BOOP), pleuritis, sarcoidosis, and asthma exacerbations can be observed during combined treatment.^2,3^ Interstitial pneumonia is among the rare side effects of IFN alpha and reported as 0.4% in the study series.^4^ Although it can occur at any phase of the treatment, it was generally observed in weeks 2 to 16 in the reported cases, and it generally manifests with dry cough and shortness of breath.^5^ In the case reported by Atug et al, interstitial pneumonia findings were detected in the 36th week of the treatment.^6^ Again in the literature, it was reported that interstitial pneumonia related to PEG IFN mostly improves with the discontinuation of the medicine; however, it was reported that it can also be unresponsive to treatment or with a fatal course.^5,6^ In our case, interstitial pneumonia emerged with the symptoms of dry cough and shortness of breath in the 28th week of the treatment, and it was observed that the findings improved with the discontinuation of the medicine. Although its mechanism is not precisely known, direct toxic effect of drug or cytotoxic T-cell activity and soluble IL-2 receptor, IL-18 binding proteins, platelet-derived growth factor, and immunomodulatory reactions, such as tumor growth factor-beta induction can help explain the effects observed.^7^

Consequently, interstitial pneumonia should be taken into consideration in the differential diagnosis of the pulmonary symptoms that may occur during PEG IFN treatment. This rare complication can have a serious course and the treatment should be stopped when necessary.
